# Association between environmental exposures and granulomatosis with polyangiitis in Canterbury, New Zealand

**DOI:** 10.1186/s13075-015-0852-6

**Published:** 2015-11-24

**Authors:** Lisa K. Stamp, Peter T. Chapman, Janine Francis, Lutz Beckert, Christopher Frampton, Richard A. Watts, John L. O’Donnell

**Affiliations:** Department of Medicine, University of Otago, Christchurch, P.O. Box 4345, Christchurch, New Zealand; Department of Rheumatology, Immunology and Allergy Christchurch Hospital, Private Bag 4710, Christchurch, New Zealand; Ipswich Hospital NHS Trust, Heath Road, Ipswich, IP4 5PD UK

**Keywords:** Granulomatous polyangiitis, Epidemiology, Environment, Exposures

## Abstract

**Introduction:**

Granulomatosis with polyangiitis (GPA) is a rare systemic vasculitis. While aetiology is unknown the prominent respiratory involvement suggests inhaled antigens may be involved. The aim of this study was to identify environmental risk factors associated with GPA in Canterbury, New Zealand.

**Methods:**

A case-controlled study was undertaken. All GPA cases fulfilled American College of Rheumatology (ACR), Chapel Hill Consensus Criteria (CHCC) or the European Medicines Agency (EMA) criteria. Each case was gender matched with four controls - 2 musculoskeletal (osteoarthritis or fracture) and 2 respiratory (asthma or chronic obstructive pulmonary disease). One musculoskeletal control and one respiratory control were age matched with the case at the time of the interview (interview) and the remaining two controls were age matched at the time their case experienced the first symptom of vasculitis (index). A structured questionnaire to assess potential environmental agents was administered without blinding for case/control status. Data were analyzed using conditional logistic regression to allow for the individual matching of cases and controls to assess for association between environmental factors and GPA.

**Results:**

49 cases and 196 controls were recruited. 53 % were male and the mean ± standard deviation (SD) age of the cases was 64.9 ± 12.4 years, interview controls 65.1 ± 12.4.years and index controls 53.9 ± 14.5 years. Any reported exposure to dust (specifically silica and grain dust) was associated with GPA, odds ratio (OR) 3.6 (95 % confidence interval (CI); 1.5–8.3, *p* = 0.003). Occupation as a farm worker was associated with GPA OR 3.43 (1.5–7.5, *p* = 0.002). Specific gardening activities were associated with GPA including digging (OR 3.2; 1.4–7.0; *p* = 0.003), mowing (OR 2.7; 1.3–5.8; *p* = 0.008) and planting (OR 2.6; 1.2–5.5; *p* = 0.013).

**Conclusion:**

We have replicated findings from northern hemisphere studies identifying dust exposure as well as farm exposure as risk factors for the development of GPA. We have shown activities associated with exposure to inhaled antigens, in particular those related to farming or gardening activities may increase the risk of GPA.

## Introduction

Granulomatosis with polyangiitis (GPA) is a systemic vasculitis affecting small vessels of the upper and lower respiratory tracts and the kidneys. GPA is usually associated with the presence of anti-neutrophil cytoplasmic antibodies (ANCA) directed against proteinase 3 (PR3). The aetiology of GPA is unknown.

While GPA is uncommon, there is significant geographical variation in the prevalence of disease. A latitudinal gradient has been identified with increasing prevalence as the distance from the equator increases [1, 2]. Exposure to specific environmental factors in genetically susceptible individuals is thought to be a key mechanism for triggering disease. The geographical variation in disease as well as the classical upper and lower respiratory tract involvement suggests that environmental factors, particularly inhalants, may have a role in aetiology. Other factors such as seasonal variation in onset and urban–rural differences in disease frequency lend support to a role for environmental factors [1].

Of particular note are exposure to silica, dust and occupation in farming, all of which may be associated with exposure to inhaled potential antigens [1, 3]. To date the studies examining the relationship between environmental factors and GPA have been undertaken in the Northern Hemisphere. The aim of the current study was to identify environmental risk factors associated with GPA in Canterbury, New Zealand (NZ), an area with a high prevalence of the disease [4].

## Methods

A case-controlled study was undertaken in Canterbury, NZ. The province of Canterbury on the South Island of NZ had a population of 539,436 at the 2013 census. Christchurch is the main urban centre within the region, where 63 % of the regional population reside. Christchurch Hospital is the major provincial referral centre and public hospital. All cases and controls were obtained from Christchurch Hospital Rheumatology, Nephrology, Orthopaedic or Respiratory Departments. Ethical approval was received from the Upper South B Regional Ethics Committee, NZ. All participants gave written informed consent.

### Cases

Patients with GPA in Canterbury, NZ were identified from databases in the Rheumatology and Nephrology Departments at Christchurch Hospital. All living patients were included up until 21 May 2012. Cases were classified as GPA according to one of the following criteria; American College of Rheumatology (ACR) [5], the Chapel Hill Consensus Conference (CHCC) definition [6] or the European Medicines Agency (EMA) criteria [7]. Case notes were reviewed for clinical and laboratory variables including the organ system involved and the presence of ANCA. Respiratory involvement was defined as pleurisy, bloody nasal discharge, haemoptysis, sinus, subglottic involvement or an abnormal chest X-ray. Renal involvement was defined by the presence of haematuria, red cell casts, proteinuria or new abnormal creatinine.

### Non-vasculitis controls

Four controls were recruited for each case. Two controls had non-inflammatory musculoskeletal disease (osteoarthritis or fracture) and were identified from orthopaedic surgical waiting lists at Christchurch Hospital. The remaining two controls had respiratory disease (chronic obstructive pulmonary disease (COPD) or asthma) and were identified from the respiratory laboratory lung function test database. These diseases were selected as controls because they have overlapping clinical features with GPA. Controls were excluded if they had a personal history of other inflammatory diseases including inflammatory arthritis, connective tissue disease or inflammatory bowel disease. Controls were age ± 5 (years) and gender matched. One non-inflammatory musculoskeletal disease and one respiratory disease control were matched to the age that cases had been when they first experienced a symptom attributable to vasculitis (index date). These participants were asked to recall events in the year immediately preceding the interview. The other non-inflammatory musculoskeletal disease and respiratory disease controls were matched to the age of the case at the time of interview and were asked to think back to events that occurred prior to the index date of the matched case. Age matching was undertaken in this manner to control for recall bias and temporal effects.

### Questionnaire

A structured questionnaire to identify environmental factors was adapted from that used by Lane et al. [3]. A single interviewer (JF) conducted all interviews either in person at Christchurch Hospital or by telephone. The interviewer was not blinded to case/control status nor to the aims of the study.

### Exposures

Silica, metal and solvent exposures were based on occupation. Occupations were coded using the Standard Occupation Classification 2000 and job exposure matrices [8] used to define high, intermediate and low exposure. Intensity of exposure was calculated as the duration of exposure (years) × level of exposure (where 1 = low, 2 = intermediate and 3 = high). A full list of exposures assessed in the questionnaire is presented in Table 1. Exposures were examined in two time frames; in the 12 months prior to the index date only, or at any time prior to the index date (lifetime exposure).

**Table 1:** Exposures assessed in the questionnaire

Exposure	Detail
Occupation	All jobs that lasted >6 months prior to the index date or interview date if control (if <25 years at index date, then all jobs >3 months)
	Specific exposure to grain and silica dust
	Specific occupation questions—farm worker, baker, brick/foundry worker, sand blaster, dental technician, mine/quarry worker
Hobbies	Any hobby regularly spent time doing prior to index date/interview and what exposures those hobbies led to
	Specific questions about gardening, chemical/fume exposure with hobby, fungus/mould exposure
Pets	Any pet ownership prior to index date
Residence history	Place of residence for 2 years prior to diagnosis
	Specific questions about type of heating—gas, oil, electricity, wood burning, coal, other
	Specific questions about construction occurring in the dwelling
Farm exposure	Four mutually exclusive categories of farm exposure—live on a farm, visit any farm, work on a farm or none.
	Farm any—indicates the individual lived, visited or worked on a farm
	Crop exposure—general question about exposure followed by specific questions about wheat, barley, oats, hay, exposure quantified as daily, weekly, monthly or less often exposure of any type
	Livestock exposure—general question about exposure followed by specific questions about cows, pigs, sheep, goats, chickens, exposure quantified as daily, weekly, monthly or less often
Allergy	Skin reactions to metals, soap, creams, other
	Allergic rhinitis
	Asthma
	Drug allergy
	Insect allergy
	Plant allergy
	Food allergy
	History of tuberculosis, hepatitis, blood transfusion
	Smoking history—duration, quantity
	Family history of allergies, vasculitis

### Data and statistical analysis

Demographic features of cases and controls are summarised using means and ranges and frequencies in percentages as appropriate. The univariate associations between putative risk factors and GPA were tested using conditional logistic regressions to allow for the individual matching of cases and controls. The strength of the associations are summarised with odds ratios (ORs) and 95 % confidence intervals (CIs).

Four categories of risk factors were identified; farming, dusts and fumes, gardening, and other. Risk factors identified within each of these categories from the univariate analyses were entered into forward and backward stepwise conditional logistic regression models. Variables that showed significant associations from both forward and backward models were considered independent risk factors. Significant independent predictors from each category were then entered into a forward and backward stepwise model to determine the final set of independent risk factors for GPA.

The prominent respiratory involvement in GPA has led to the suggestion that inhaled antigens may trigger disease and therefore risk factors may differ between those with and without respiratory involvement. To determine whether the strength of the associations between the cases with and without respiratory involvement was significantly different we generated a set of additional conditional logistic regression models. These models, as well as including the main effects of cases vs. controls and the risk factor, also included an interaction term between these two effects. This interaction term tests whether the ORs for those with and without respiratory disease are significantly different.

Two-sided *p* <0.05 was taken to indicate statistical significance. No correction for multiple comparisons was undertaken. All statistical analyses were undertaken using SPSS v21.0 (IBM Software).

## Results

Forty-nine cases and 196 controls were interviewed between 16 June 2010 and 10 January 2013. Only one patient with GPA declined to participate. Eighteen OA controls (mean age 63.8 years (range 30–89), 66.7 % female) and 16 COPD controls (mean age 66.7 years (range 49–87), 43.4 % female) declined to participate. Demographic details of the cases and controls are presented in Table 2. No GPA patients died during the study period. Of the GPA cases, 61.2 % fulfilled the ACR criteria, 97.9 % fulfilled the EMA criteria and 42.9 % fulfilled the CHCC criteria. ANCA was positive in 48/49 (97.9 %) cases and PR3 antibodies were positive in 43/48 (89.6 %). Of the five PR3-negative patients, three were MPO-positive and two were MPO-negative; in one patient, PR3 and MPO were not determined. The most common clinical features at presentation were arthritis/arthralgia (65 %), sinusitis (61 %) and renal involvement (63 %). Respiratory involvement occurred in 38/49 (77.6 %). Only 20 % of patients had haemoptysis. Mean disease duration for the GPA cases was 11.3 years (range 0.1–31.0 years).

**Table 2:** Demographics of the patients with GPA and controls^a^

	GPA	COPD index	COPD interview	OA index	OA interview
	(*n* = 49)	(*n* = 49)	(*n* = 49)	(*n* = 49)	(*n* = 49)
Age at interview (years)	64.9 (33–89)		65.1 (33–87)		65.1 (31–87)
Age at index date (years)	53.3 (20–81)	54.3 (23–83)		53.6 (22–81)	
Male	26 (53.1 %)	26 (53.1 %)	26 (53.1 %)	26 (53.1 %)	26 (53.1 %)
NZ European/European	45 (91.8 %)	46 (93.9 %)	46 (93.9 %)	48 (97.9 %)	46 (93.9 %)
NZ Maori	4 (8.2 %)	3 (6.1 %)	3 (6.1 %)	1 (2 %)	3 (6.1 %)
Ever smoker	28 (57.1 %)	38 (77.6 %)	44 (89.8 %)	25 (51.0 %)	23 (46.9 %)

Data presented as mean (range) or number (%)

^a^The column headed GPA describes the cases, while the columns headed COPD and OA describe the two sets of disease control. Disease controls were further divided into those age-matched ±5 years for age at onset of disease (index) and those matched for age at year of interview (interview)

*COPD* chronic obstructive pulmonary disease, *GPA* granulomatosis with polyangiitis, *NZ* New Zealand, *OA* osteoarthritis

### Farming

Living or working on a farm at any time prior to the index date was not associated with GPA (Table 3). However, living, visiting or working on a farm in the 12 months prior to the index date was associated with GPA (OR 2.74 (95 % CI 1.3–5.9)). Examining each category of exposure (living, visiting or working) revealed that only visiting a farm was associated with GPA (Table 3).

**Table 3:** Associations between farming and GPA

	GPA	Controls	OR (95 % CI)	*p* value
Farm live or work (not visit) at any time prior to index date	22 (44.9 %)	69 (35.2 %)	1.7 (0.8–3.4)	0.16
Farm any^a^ vs. none in the 12 months prior to index date	23 (46.9 %)	58 (29.6 %)	2.7 (1.3–5.8)	0.009
Farm visit vs. everything else (work, live, none) in the 12 months prior to index date	16 (32.7 %)	23 (11.7 %)	7.2 (2.6–19.4)	<0.001
Farm live vs. everything else (work, visit, none in the 12 months prior to index date	6 (12.2 %)	24 (12.2 %)	1 (0.3–2.9)	1
Farm work vs. everything else (live, visit, none) in the 12 months prior to index date	1 (2.0 %)	11 (5.6 %)	0.3 (0.03–2.6)	0.26
Crops in the 12 months prior to index date (could include livestock)	18 (36.7 %)	54 (27.6 %)	1.7 (0.8–3.6)	0.16
Livestock in the 12 months prior to index date (could include crops)	29 (59.2 %)	85 (43.4)	2.4 (1.1–5.0)	0.02
Crops and livestock (vs. none or exposure to one)	16 (32.7 %)	53 (27.0 %)	1.4 (0.7–2.97	0.39
Crops and livestock vs. no livestock (i.e. includes crops only and excludes livestock only)	16/36 (44.4 %)	53/164 (32.3 %)	2.20 (0.9–5.5)	0.09
Cows	23 (46.9 %)	73 (37.2 %)	1.6 (0.8–3.3)	0.17
Pigs	13 (26.5 %)	51 (26.0)	1.0 (0.5–2.3)	0.94
Horses	18 (36.7 %)	49 (25.0 %)	1.9 (0.93–.9)	0.08
Sheep	26 (53.1 %)	60 (30.6 %)	3.6 (1.7–7.7)	0.001
Chickens	18 (36.7 %)	51 (26.0 %)	2.0 (0.9–4.4)	0.08

^a^This category includes all those who lived, worked or visited a farm in the 12 months prior to the index date. The individual categories live, work or visit were mutually exclusive

*CI* confidence interval, *GPA* granulomatosis with polyangiitis, *OR* odds ratio

Most individuals were exposed to crops and livestock. There was a significant association between exposure to livestock and GPA (Table 3). For individual types of livestock there was a significant association with sheep but not cows, pigs or chickens (Table 3). Those reporting their occupation as a farm worker were also at increased risk of GPA (OR 3.4 (95 % CI 4.6–7.6); *p* = 0.002).

### Dust and fumes exposure

Exposure to any dusts as well as specific exposure to grain or silica dust was associated with GPA (Table 4). Exposure to chemical sprays, glue/paint fumes and petrol/diesel fumes was based on hobbies identified by the participants. GPA was associated with exposure to chemical sprays, petrol and diesel fumes (Table 4).

**Table 4:** Dust exposure and risk of GPA

	GPA	Controls	OR (95 % CI)	*p* value
Any dust	27 (55.1 %)	69 (35.2 %)	3.6 (1.6–8.3)	0.003
Grain dust	23 (46.9 %)	51 (26 %)	4.2 (1.8–9.9)	0.001
Silica dust	16 (32.7 %)	36 (18.4 %)	3.1 (1.3–7.4)	0.013
Chemical sprays	6 (12.2 %)	9 (4.6 %)	5.3 (1.3–21.7)	0.02
Petrol/diesel fumes	7 (14.3 %)	11 (5.6 %)	3.8 (1.1–13.1)	0.03

*CI* confidence interval, *GPA* granulomatosis with polyangiitis, *OR* odds ratio

Working in occupations with high silica, solvent or metal exposure in the year prior to the index date was not associated with GPA (*p* >0.05 for all). As already noted, intensity of exposure was calculated as the duration of exposure (years) multiplied by the level of exposure (where 1 = low, 2 = intermediate and 3 = high based on occupation) over the individual’s working lifetime. GPA was associated with higher mean intensity of lifetime occupational exposure prior to the index date for silica (*p* = 0.001), solvents (*p* = 0.001) and metals (*p* = 0.003).

### Gardening

In the year prior to the index date, those with GPA spent significantly more time in the garden (22.7 ± 4.1 hours/month vs. 13.2 ± 2.0; *p* = 0.04). Specific gardening activities were associated with GPA including digging (OR 3.2 (95 % CI 1.4–7.0); *p* = 0.003), mowing (OR 2.8 (95 % CI 1.3–5.8); *p* = 0.008) and planting (OR 2.6 (95 % CI 1.2–5.6); *p* = 0.013). Other activities were not associated with GPA including pruning (OR 1.84 (95 % CI 0.89–3.82); *p* = 0.1), and weeding (OR 1.56 (95 % CI 0.76–3.21); *p* = 0.23) (Table 5).

**Table 5:** Gardening exposures in cases and controls

	GPA	Controls	OR (95 % CI)	*p* value
Gardening	41 (83.7)	140 (71.4)	2.40 (0.98–5.85)	0.054
Digging	23 (46.9 %)	54 (27.6 %)	3.22 (1.48–7.00)	0.003
Pruning	22 (44.9 %)	66 (33.7 %)	1.84 (0.89–3.82)	0.101
Mowing	29 (59.2 %)	80 (40.8 %)	2.76 (1.31–5.81)	0.008
Planting	31 (63.3 %)	90 (45.9 %)	2.61 (1.22–5.57)	0.013
Weeding	34 (69.4 %)	119 (60.7 %)	1.56 (0.76–3.21)	0.227
Spraying	5 (10.2 %)	13 (6.6 %)	1.78 (0.53–5.99)	0.35
Time spent gardening per month (hours)	22.7 ± 4.1	13.2 ± 2.0		0.037

*CI* confidence interval, *OR* odds ratio, GPA granulomatosis with polyangiitis

### Other exposures

There was no association between a personal or family history of allergy and GPA (Table 6). There was no association between GPA and smoking (OR 0.59 (95 % CI 0.28–1.25); *p* = 0.17), previous blood transfusions (OR 0.57 (95 % CI 0.20–1.68); *p* = 0.31), hepatitis (OR 1.31 (95 % CI 0.36–4.79); *p* = 0.68) or tuberculosis (OR 0.37 (95 % CI 0.04–3.43); *p* = 0.38) (Table 6). Vaccination within 6 months of the index date was associated with a reduced risk of GPA (OR 0.4 (95 % CI 0.2–0.9); *p* = 0.03). No significant associations between pet ownership or type of home heating, including central heating, wood burner and coal, and GPA were identified (Table 6).

**Table 6:** Association between allergy, smoking and other exposures and GPA

	GPA	Controls	OR (95 % CI)	*p* value
Any allergy	41 (83.7 %)	152 (77.6 %)	1.58 (0.65–3.85)	
Skin reactions	7 (14.3 %)	42 (21.4 %)	0.54 (0.20–1.41)	0.21
Allergic rhinitis	23 (46.9 %)	84 (42.9 %)	1.22 (0.61–2.45)	0.57
Asthma	24 (49.0 %)	109 (55.6 %)	0.73 (0.37–1.45)	0.37
Drug allergy	10 (20.4 %)	53 (27.0 %)	0.62 (0.26–1.47)	0.28
Insect allergy	7 (14.3 %)	15 (7.7 %)	2.38 (0.80–7.09)	0.15
Plant allergy	12 (24.5 %)	49 (25.0 %)	0.96 (0.42–2.23)	0.94
Food allergy	9 (18.4 %)	26 (13.3 %)	1.66 (0.63–4.37)	0.31
Tuberculosis	1 (2.0 %)	9 (4.6 %)	0.37 (0.04–3.43)	0.38
Hepatitis	4 (8.2 %)	13 (6.6 %)	1.31 (0.36–4.79)	0.68
Blood transfusion	6 (12.2 %)	34 (17.3 %)	0.57 (0.20–1.68)	0.31
Ever smoked	28 (57.1 %)	130 (66.3 %)	0.59 (0.28–1.25)	0.17
Family history of allergy	32 (65.3 %)	126 (64.3 %)	1.05 (0.52–2.14)	0.89
Vaccination	12 (24.5 %)	77 (39.3 %)	0.41 (0.19–0.92)	0.031

Data presented as number (%)

*CI* confidence interval, *GPA* granulomatosis with polyangiitis, *OR* odds ratio

### Multivariate analysis

Multivariate analyses were conducted within four exposure categories (farming, dusts and fumes, gardening, and other) to determine important independent associations (Table 7). A final model was generated which included all variables significant in the previous four models. This revealed an increased risk of GPA with exposure to grain dust (OR 4.0 (95 % CI 1.1–13.8)), hobby dust (OR 8.9 (95 % CI 2.4–28.3)), farm worker (OR 3.5 (95 % CI 1.1–11.5)), visiting a farm (OR 8.9 (95 % CI 2.6–29.9)), petrol/diesel fumes (OR 6.2 (95 % CI 1.2–32.7) and weaker association with solvent exposure (OR 1.04 (95 % CI 1.02–1.07)). There was no significant difference between controls age matched at interview or age matched at the index date for any of the variables independently associated with GPA in the final model (all *p* >0.25).

**Table 7:** Multivariate analysis of variables associated with GPA

Model	Variables included	Significant variables (OR (95 % CI))	
Gardening	Digging, planting, mowing and time in garden	Digging	2.6 (1.2–5.7)
		Mowing	2.3 (1.0–5.0)
Farming	Farm worker, livestock, sheep, farm any in 12 months prior to index date, farm visit in 12 months prior to index date	Farm visit	9.3 (3.2–26.7)
		Farm worker	4.4 (1.9–10.3)
Dusts	Any dust, grain dust, silica dust, chemical sprays, construction, petrol/diesel fumes, hobby dust silica, solvent, metals	Grain dust	7.2 (2.7–19.4)
		Hobby dust	8.1 (2.6–24.7)
		Petrol/diesel	5.1 (1.2–19.9)
		Solvent exposure	1.03 (1.01–1.05)
Vaccination	Vaccination		0.4 (0.2–0.9)
Combined	Digging, mowing, farm visit, farm worker, grain dust, hobby dust, petrol/diesel fumes, solvent exposure, vaccination	Grain dust	4.0 (1.1–13.8)
		Hobby dust	8.3 (2.4–28.3)
		Farm worker	3.5 (1.1–11.5)
		Farm visit	8.9 (2.6–29.9)
		Petrol/diesel	6.2 (1.2–32.7)
		Solvent exposure	1.04 (1.02–1.07)

*CI* confidence interval, *GPA* granulomatosis with polyangiitis, *OR* odds ratio

### Respiratory involvement

The potential for inhaled antigens features strongly in the current analysis. Therefore we compared the ORs between those GPA cases with (*n* = 38) and without (*n* = 11) respiratory involvement, defined as any one of the following clinical features: pleurisy, bloody nasal discharge, haemoptysis or sinus involvement. Of the significant univariate risk factors, only the ORs between mowing and farm work were significantly different between those with and without respiratory involvement; mowing (OR 8.8 (95 % CI 1.3–59.0)) and farm work (OR 0.03 (95 % CI 0.002–0.59)). Interestingly, there was a lower risk of respiratory involvement in those with GPA who were farm workers when compared with those who were not farm workers.

A multivariate analysis of all significant variables in previous models (i.e. digging, farm visit, farm worker, grain dust, hobby dust, petrol/diesel fumes and solvent exposure) was undertaken only in those with respiratory involvement. Similar associations were observed as in the whole group with an increased risk of GPA independently associated with hobby dust (OR 11.5 (95 % CI 3.1–43.3)), visiting a farm (OR 9.9 (95 % CI 2.7–36.5)), petrol/diesel fumes (OR 5.6 (95 % CI 1.0–31.3)) and weakly with solvent exposure (OR 1.04 (95 % CI 1.00–1.06)).

## Discussion

We have replicated studies from the Northern Hemisphere that identified a number of environmental risk factors associated with GPA. Inhaled factors feature in the current analysis including dusts, and fumes as well as activities that may lead to exposure to inhaled antigens such as digging in the garden and being a farm worker.

Farm exposure has previously been associated with an increased risk of GPA [3], although this is not a universal finding [9]. Of particular note, both the current study and the previous study by Lane et al. [3] reported an increased risk of GPA in those visiting a farm in the 12 months prior to the index date but not in those living on a farm. Association between working on a farm and GPA was not consistent between the two studies. One potential explanation for these observations is that individuals living on farms are tolerised to the environmental ‘antigen’ by virtue of their early, more long-term exposure, while later exposure in those visiting or working on a farm predisposes to an aberrant immunological response and subsequent disease. Both studies also reported an association between GPA and livestock exposure, although associations with specific types of animals differed between the countries; sheep in NZ and cows/chickens in the UK. This difference in type of livestock exposure most probably reflects the local farming practices of the two countries. The numbers are small and most individuals were exposed to more than one type of animal, making definitive conclusions about the observed relationships difficult.

Unlike other studies we observed a relationship between some gardening activities and GPA. The specific gardening activities (digging, mowing and planting), which were associated with GPA, may all result in potential antigens being distributed into the local air space from where they could be inhaled. A number of different pathogens are present in soil, particularly bacterial and fungal species. Thus it is conceivable that the common link between gardening and farming activities relates to the exposure to microbial species.

One potential mechanism whereby microbes might trigger auto-immune disease is through the production of auto-antibodies. Of particular relevance for GPA are autoantibodies to human lysosomal membrane protein-2 (hLAMP-2), which have been reported to be present in patients with ANCA associated pauci-immune focal necrotising glomerulonephritis (FNGN) [10, 11]. hLAMP-2 has sequence homology with the bacterial human virulence determinant known as FimH [11] which survives in airborne soil particles [12]. The type and quantity of microbes present in soil can vary across geographical regions depending on soil pH, temperature, UV light exposure, moisture and nutrients available [13].

We have also shown that exposure to dusts and silica may be associated with an increased risk of GPA. Silica exposure has been associated with a number of auto-immune diseases including GPA [14], rheumatoid arthritis [15] and systemic lupus erythematosus [16]. The mechanisms whereby silica results in auto-immunity are not fully understood but are thought to include its potential action as a non-specific immune adjuvant for activation of responder T cells and regulatory T cells (Tregs) [17]. We have shown a relationship between the intensity of exposure and disease. Further clarification of the timing of exposure as well as the threshold of intensity or duration of exposure for the risk of disease to increase will require long-term prospective studies.

The strengths of the current study include the large number of GPA cases included. We are confident that case ascertainment was complete and only one GPA patient declined to participate. The current study has a number of limitations. Although this study may be considered small, it is a relatively large study given how rare GPA is. Recall bias by both cases and controls is unavoidable in any retrospective study. However, both cases and controls were asked to recall a similar time period in relation to the ‘index date’ in an effort to minimise recall bias and temporal effects. It is also possible that the selection of disease controls (i.e. patients with osteoarthritis/fracture/COPD/asthma) may also have introduced a source of bias. Controls with these conditions may have been less likely to have physical jobs such as farming or undertake physical activities such as gardening. However, participants were asked to consider events related to the index date some years before when the presence or effects of these conditions was likely to have been less. The controls with COPD were also more likely to have been smokers; indeed, 85 % of COPD patients were current smokers or ex-smokers. This most probably accounts for the apparent protective effect of smoking, albeit non-significant. In addition, more controls declined to participate which may have introduced an element of selection bias. We were unable to confirm socio-economic status (SES) and differences in SES may influence hobbies, residence and the ability to visits farms. Finally, the interviewer was not blinded to the case/control status of the participants and there was heterogeneity of the interview method. While the vast majority of cases and COPD controls were interviewed face to face during scheduled hospital attendances, most of the OA controls were interviewed by telephone.

## Conclusions

This case–control study has largely replicated the findings from the Northern Hemisphere investigating the environmental risk factors for GPA. A common link between the risk activities could be exposure to airborne particles. It is possible that inhaled bacterial antigens such as FimH within such particles are aetiopathogeneic. The exact mechanism whereby inhaled antigens might contribute to disease remains unclear, particularly since GPA is a rare disease and the inhaled environmental exposures are relatively common, suggesting that other factors must be important. Further study is required to elucidate these mechanisms.

## Abbreviations

ACR: American College of Rheumatology
ANCA: Anti-neutrophil cytoplasmic antibodies
CHCC: Chapel Hill Consensus Criteria
CI: Confidence interval
COPD: Chronic obstructive pulmonary disease
EMA: European Medicines Agency
FNGN: Focal necrotising glomerulonephritis
GPA: Granulomatosis with polyangiitis
hLAMP-2: Human lysosomal membrane protein-2
NZ: New Zealand
OR: Odds ratio
PR3: proteinase 3
SD: Standard deviation
SES: Socio-economic status
Treg: regulatory T cell
